# TMEM9B Regulates Endosomal ClC-3 and ClC-4 Transporters

**DOI:** 10.3390/life14081034

**Published:** 2024-08-20

**Authors:** Margherita Festa, Maria Antonietta Coppola, Elena Angeli, Abraham Tettey-Matey, Alice Giusto, Irene Mazza, Elena Gatta, Raffaella Barbieri, Alessandra Picollo, Paola Gavazzo, Michael Pusch, Cristiana Picco, Francesca Sbrana

**Affiliations:** 1 Istituto di Biofisica, Consiglio Nazionale delle Ricerche, 16149 Genova, Italy; margherita.festa@ibf.cnr.it (M.F.); mariaantonietta.coppola@ibf.cnr.it (M.A.C.); abraham.matey@ibf.cnr.it (A.T.-M.); alice.giusto@ibf.cnr.it (A.G.); irene.mazza@ibf.cnr.it (I.M.); raffaella.barbieri@ibf.cnr.it (R.B.); alessandra.picollo@ibf.cnr.it (A.P.); paola.gavazzo@ibf.cnr.it (P.G.); 2 DIFI Lab, Dipartimento di Fisica, Università di Genova, 16146 Genova, Italy; elena.angeli@unige.it (E.A.); elena.gatta@unige.it (E.G.)

**Keywords:** ClC-3, ClC-4, TMEM9B, endosomes, neurodevelopmental disorders, subunit

## Abstract

The nine-member CLC gene family of Cl^−^ chloride-transporting membrane proteins is divided into plasma membrane-localized Cl^−^ channels and endo-/lysosomal Cl^−^/H^+^ antiporters. Accessory proteins have been identified for ClC-K and ClC-2 channels and for the lysosomal ClC-7, but not the other CLCs. Here, we identified TMEM9 Domain Family Member B (TMEM9B), a single-span type I transmembrane protein of unknown function, to strongly interact with the neuronal endosomal ClC-3 and ClC-4 transporters. Co-expression of TMEM9B with ClC-3 or ClC-4 dramatically reduced transporter activity in *Xenopus* oocytes and transfected HEK cells. For ClC-3, TMEM9B also induced a slow component in the kinetics of the activation time course, suggesting direct interaction. Currents mediated by ClC-7 were hardly affected by TMEM9B, and ClC-1 currents were only slightly reduced, demonstrating specific interaction with ClC-3 and ClC-4. We obtained strong evidence for direct interaction by detecting significant Förster Resonance Energy Transfer (FRET), exploiting fluorescence lifetime microscopy-based (FLIM-FRET) techniques between TMEM9B and ClC-3 and ClC-4, but hardly any FRET with ClC-1 or ClC-7. The discovery of TMEM9B as a novel interaction partner of ClC-3 and ClC-4 might have important implications for the physiological role of these transporters in neuronal endosomal homeostasis and for a better understanding of the pathological mechanisms in *CLCN3-* and *CLCN4*-related pathological conditions.

## 1. Introduction

CLC proteins are members of a nine-member gene family of anion transporting proteins, of which four (ClC-1, ClC-2, ClC-Ka, ClC-Kb) are plasma-membrane localized chloride channels, while the remaining five members (ClC-3 to -7) are endo-/lysosomal secondary active chloride proton exchangers [1]. Structurally, all CLC-proteins are dimers with physically separate ion transport pathways in each subunit [1,2,3,4,5]. The physiological relevance of CLC proteins is underscored by their involvement in various human genetic diseases, ranging from myotonia (ClC-1), kidney diseases (ClC-K, ClC-5), and osteopetrosis (ClC-7) to hyperaldosteronism (ClC-2), neurodevelopmental delay (ClC-3, ClC-4, ClC-6), and neurodegeneration (ClC-6, ClC-7) [1,6,7,8].

The endosomal transporters ClC-3, -4, and -5 are structurally very similar to each other sharing about 80% sequence identity [1]. These proteins aid in the endosomal acidification process [9] and/or are involved in luminal chloride regulation [1]. While ClC-5 is kidney-specific, ClC-3 and ClC-4 are most important in the brain, as evidenced by the neurodevelopmental delay caused by mutations in the genes encoding them [7,10,11,12,13]. Interestingly, several disease variants of *CLCN3* and *CLCN4,* the genes encoding ClC-3 and ClC-4, respectively, showed unaltered functional activity in heterologous expression systems [7,12]. A possibility to explain the pathogenicity of such variants is that they might affect the interaction with so far unknown interacting proteins.

In fact, many membrane transport proteins are associated with one or more accessory subunits, which are either integral membrane proteins or soluble intra- or extracellular proteins. Accessory proteins can affect cellular trafficking, targeting, protein stability, and functional properties; they can be tissue-specific and are generally of fundamental physiological and pathophysiological importance. For members of the CLC family, accessory subunits have been discovered so far for kidney ClC-K channels, which associate with the two transmembrane domain protein barttin in an obligate manner [14]; ClC-7, which requires the one transmembrane domain protein Ostm1 for function and protein stability [15], and ClC-2, which associates with GlialCAM in astrocytes and oligodendrocytes, but not in other cells [16]. No accessory subunits have been described for the muscle channel ClC-1 or the endosomal transporters ClC-3, -4, -5, and -6 [1].

Motivated by the appearance of the small transmembrane protein of unknown function TMEM9B in interaction databases (https://thebiogrid.org (accessed on 7 August 2023)), we discovered that TMEM9B strongly interacts with endosomal ClC-3 and ClC-4 chloride/proton antiporters, but not with the lysosomal ClC-7, and only slightly with the muscle channel ClC-1. Co-expression of TMEM9B with ClC-4 suppresses its function, and co-expression with ClC-3 additionally induces a slowing of activation kinetics. Our results open a new avenue of research into the biology of endolysosomal CLC exchangers.

## 2. Materials and Methods

### 2.1. Plasmids

C-terminally green fluorescent protein (GFP)-tagged wildtype human ClC-1, -4, and -7 and mouse ClC-3c pEGFP (Clontech) and C-terminally red fluorescent (mCherry)-tagged wildtype mouse TMEM9B pFROG [17] constructs were produced in-house (by molecular cloning the sequences into the respective plasmids). Briefly, the SGLRSREF-GFP tag sequence was added at the C-terminus of both ClC-3c and ClC-4 open reading frame sequences amplified from a *Xenopus* oocyte expression vector, pTLN [18], and placed in pEGFP by Gibson Assembly [19] using the GeneArt Gibson Assembly Kit (Thermo Fisher Scientific, Milan, Italy) to obtain ClC-3c and -4 pEGFP constructs. Plasmids expressing GFP-tagged ClC-1 and -7 proteins in mammalian cells were previously described [20,21], and ClC-3c and ClC-4 expression sequences in the *Xenopus* oocyte system were also previously described [7,12].

The *Mus musculus* TMEM9B DNA open reading frame was amplified from pLV Tmem9b-V5 (Addgene plasmid #175152) using the following primers: TMEM9B-HindIII-F: atgaagcttatggcgagcctatggtgcgga and TMEM9B-NotI-R: tggcggccgcgctgaggacaacgtgtcgg. The PCR product was digested with HindIII and NotI restriction enzymes and ligated into pFrog-mCherry [20], already opened with the same enzymes and dephosphorylated, to obtain the TMEM9B-mCherry-pFrog plasmid. Another version with EGFP as C-terminally fused fluorophore was prepared substituting mCherry by a GFP-6xHis-TAG from another vector using NotI and EcoRI enzymes. The TMEM9B sequence was also subcloned in the pTLN vector [18], suitable for *Xenopus* oocyte expression. All constructs were verified by Sanger sequencing.

### 2.2. HEK Cell Culture and Transfection

HEK293T cells (for short HEK cells) were cultured in high glucose Dulbecco’s Modified Eagle’s Medium supplemented with 1% glutamine, 1% penicillin/streptomycin, and 10% Fetal Bovine Serum (all from Euroclone, Milan, Italy), at 37 °C, 5% CO_2_. Cells were transfected using the Effectene Kit (Qiagen, Hilden, Germany), following the manufacturer’s instructions. Briefly, cells were plated in 1% poly-L-lysine (Merck, Milan, Italy) pre-coated 35 mm plastic dishes or glass-bottom dishes (IBL Baustoff + Labor GmbH, Gerasdorf, Austria) one day before transfection at 70% confluency. They were transfected with either wildtype ClC-1-GFP, ClC-3c-GFP, ClC-4-GFP, or ClC-7-GFP alone or co-transfected with TMEM9B-mCherry. A total of 450 ng of ClC and 150 ng of TMEM9B plasmid were used in the transfection. ClC-4 was also co-transfected with TMEM9B with opposite fluorescent tags. The transfected cells in the plastic and glass-bottom dishes were, respectively, used for electrophysiological and FLIM-FRET confocal microscopy measurements, 24 h post-transfection.

### 2.3. In Vitro cRNA Synthesis

For in vitro cRNA synthesis and oocyte expression, the respective pTLN plasmids were linearized with MluI (Thermo Fisher Scientific, Milan, Italy). The linearized DNAs were checked on agarose gel. Capped cRNA was synthesized using the mMESSAGE mMACHINE SP6 Transcription Kit (Thermo Fisher Scientific, Milan, Italy) following the manufacturer’s instructions. cRNA was verified on agarose gels and stored at −20 °C.

### 2.4. Oocyte Expression

Adult female *Xenopus laevis* frogs were anesthetized by immersion in *tricaine* methanesulfonate (Merck, MS-222) for 30 min. Oocytes were surgically removed and treated with collagenase (1 mg/mL) for 1 h at 18 °C under constant shaking in a “0 Ca^2+^” solution composed of 94 mM NaCl, 1 mM KCl, and 10 mM Hepes-Na (pH 7.5). They were then washed twice and further kept in standard maintaining solution composed of 90 mM NaCl, 2 mM KCl, 1 mM CaCl_2_, 1 mM MgCl_2_, and 10 mM Hepes (pH 7.5). Isolated oocytes were injected or co-injected with 5 ng ClC-1, 8 ng ClC-3, or 6 ng ClC-4 ± 0.3 ng TMEM9B cRNA using a Drummond “Nanoject” microinjector (50 nl/oocyte). A comparison was made on the same batch of oocytes, from the same frog, and always on the same day from the injection. Oocytes were incubated at 18 °C in standard solution (supplemented with 0.1 mg/mL gentamicin) for at least 24 h before voltage-clamp experiments.

### 2.5. Two-Electrode Voltage-Clamp

Two microelectrode voltage-clamp recordings of ClC-1, ClC-3, and ClC-4, with or without TMEM9B, were performed in *Xenopus* oocytes at room temperature (20–22 °C) in a solution containing 100 mM NaCl, 10 mM Hepes, and 5 mM MgSO₄ (pH 7.3). All recordings were acquired using the freeware acquisition program GePulse (http://users.ge.ibf.cnr.it/pusch/programs-mik.htm (accessed on 1 August 2023)).

Voltage-clamp stimulation protocols were adjusted for the different CLC proteins studied, taking into account their different biophysical properties [1,22].

To measure ionic currents of ClC-3 and ClC-4, with and without TMEM9B, 19 pulses of 10 ms duration, with a voltage decrement of 10 mV from +170 mV to −10 mV, were applied from a holding potential of −30 mV. The same protocol was used for non-injected oocytes. Resulting currents were subjected to baseline subtraction and subtraction of currents from non-injected oocytes. Currents were normalized to the value obtained at 170 mV for the respective WT without TMEM9B. Data are averages from independent injections, 5 for ClC-4 and 3 for ClC-3, with at least 6 oocytes for each injection per construct. For clarity, residual capacitive artifacts were cut off in the figures.

The recording protocol for ClC-1 involved a 100 ms pulse to +60 mV, followed by 13 pulses of 200 ms with a voltage increment of 20 mV, ranging from −160 mV to +80 mV; then, a 100 ms pulse to −100 mV was applied. The membrane potential was held at −40 mV between the application of voltage-clamp pulses. Data are averages from three injections with at least 6 oocytes each. The expression level was quantified as the slope conductance of the steady state current-voltage relationship in the voltage range −40 mV ≤ V ≤ 40 mV, corresponding to the maximal slope. For ClC-1, this is the most robust method for current quantification [20].

Oocyte experiments were performed between 24 and 48 h after injection.

### 2.6. Whole-Cell Patch Clamp

Whole-cell current recordings were performed using the patch clamp technique as described previously [23]. Recording pipettes were pulled from borosilicate capillaries (Hilgenberg, Malsfeld, Germany), and filled with a standard intracellular solution containing (in mM) 130 NaCl; 10 Hepes, 2 MgCl_2_, and 2 EGTA, pH 7.3. In standard extracellular solution (in mM: 160 NaCl, 10 Hepes, 4 MgSO_4_, pH 7.3), pipette resistances ranged between 2–4 MΩ. A 3 M KCl/1% agar bridge served as ground.

Data were recorded by an Axopatch 200 amplifier (Molecular Devices), sampled at 100 kHz after filtering at 10 kHz by the amplifier-built-in 8-pole Bessel. Data were acquired using GePulse employing a National Instruments, Austin, Texas, USA interface (NI PCI-6036), and analysis was performed with Ana, programs freely available from http://users.ge.ibf.cnr.it/pusch (accessed on 1 August 2023). Prism (GraphPad) was used to perform additional analysis as needed.

Voltage protocols used to acquire transport currents were described previously [24], and appear as figure insets. To detect ClC-3 and ClC-4 transport currents, 5 ms pulses were applied from a holding potential, V_hold_, of 0 mV; these pulses progressed from 200 to −40 mV in 10 mV decrements. A *P*/*N* subtraction procedure corrected for capacitive and leak currents within raw measurements. This entailed the application of a scaled-down measurement protocol (0.2×), followed by suitable scaling and subtraction of the resultant currents from the raw currents. Additionally, to investigate the slowed activation induced by TMEM9B, from the 0 mV holding potential, we delivered 500 ms activating test pulses to positive voltages ranging between 140 mV to −80 mV. Activating pulses progressively hyperpolarized in decrements of 20 mV before applying a constant −80 mV tail pulse. Transport ClC-7 currents were measured using the same voltage protocol.

### 2.7. FLIM-FRET and Fluorescence Confocal Imaging

FLIM-FRET and fluorescence confocal imaging data were collected using a Stellaris 8 Falcon τ-STED microscope (Leica Microsystems, Mannheim, Germany) equipped with a supercontinuum pulsed (80 MHz) white light laser 440–790 nm. An HC PL APO CS2 100×/1.40 oil immersion objective lens was employed. For FLIM-FRET measurements, the donor fluorophore (GFP) was excited at a wavelength of 488 nm, and its emission was detected at 502–558 nm using a hybrid detector (Leica Microsystems, Mannheim, Germany) in photon counting mode.

We simultaneously acquired intensity and lifetime data by scanning the laser with a pixel dwell-time of 3.16 μs/pixel and 16-line accumulation. The frame size was set to 512  ×  512 pixels. For fluorescence confocal imaging, frames of 1024 × 1024 pixels were acquired, and magnification of 2× and 3× were used, corresponding to pixel sizes of 57 and 38 nm, respectively. For GFP imaging, the same excitation and emission range as those for FLIM-FRET were employed. mCherry was excited with a 561 nm laser and its emission was detected in the 571–628 nm range. LysoTracker™ Deep Red (Thermo Fisher Scientific, Milan, Italy) and CellMask™ Plasma Membrane DeepRed (Thermo Fisher Scientific, Milan, Italy) were both excited at 649 nm and emission was collected in the range 659–710 nm. All images were acquired using hybrid detectors (Leica Microsystems, Mannheim, Germany) in counting mode with a pixel dwell-time of 0.86 µs and 10-line accumulation. A transmitted light detector was used to collect bright field images.

Lifetime values were obtained using the fit-free phasor-plot approach described in [25]. Briefly, in this method, fluorescence lifetime data are Fourier transformed to get, pixel by pixel, the phasor coordinates G and S, corresponding to the real and imaginary components, respectively. The phasor plot is a heat map in the G-S plane, where the intensity is proportional to the number of pixels of the original picture (or within a selected region of interest) with corresponding G-S values. The fluorescence lifetime was determined by considering the G-S values with the largest number of associated pixels (using a Gaussian fit) and calculating the lifetime as:

$$\tau =\frac{S}{2\pi fG}$$

where f = 80 MHz is the laser pulse frequency.

The FLIM-FRET data analysis, based on the phasor plot approach, was performed using the custom AnaVision software (version 01-08-2023) (http://users.ge.ibf.cnr.it/pusch/programs-mik.htm (accessed on 1 August 2023)). The regions of interest used in the FLIM-FRET analysis were confined to the plasma membrane, or to intracellular structures exhibiting a medium level of fluorescence avoiding regions of evident agglomeration.

FRET efficiency depends on the spectral overlap between the donor emission spectrum and the acceptor absorption spectrum, the distance, and the relative orientation of the donor and the acceptor. FRET can be conveniently measured via the reduction of the donor lifetime from its value τ_D_ in the absence of the acceptor to τ_DA_ in the presence of the acceptor:

$$E=1-\frac{\tau_{DA}}{\tau_{D}}$$

The lifetime of the unquenched donor, τ_D_, was determined in each experiment from lifetime images of donor-only transfected cells. Confocal image analysis and quantification of co-localization were performed using Fiji software (https://imagej.net/ij/ (accessed on 1 August 2023)). The Coloc2 plugin was employed to determine the Pearson correlation coefficient, with default parameters applied. Fluorescence data were pooled from at least three transfections.

### 2.8. Data Representation and Statistics

Data are reported as mean values ± the standard deviation (SD) or ± standard error of the mean (SEM) as indicated in the figure legends. Tests of significance apply unpaired Student’s *t*-tests assuming equal variance.

## 3. Results

### 3.1. Identification of TMEM9B as a Candidate Interactor of CLC Proteins

**Figure 1:**
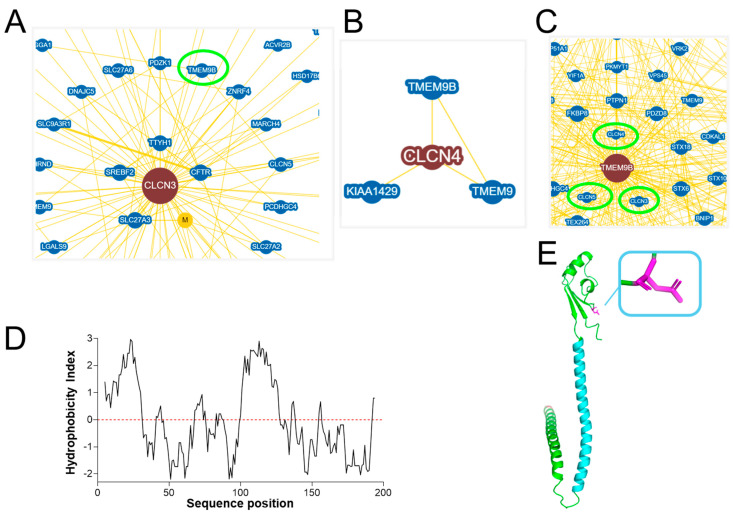
TMEM9B as a putative interactor of ClC-3 and ClC-4. (**A**). Network of ClC-3 interactors, among which is TMEM9B, from the BioGrid 4.4 database [28]. The green circle highlights the TMEM9B entry. (**B**). Network of ClC-4 interactors, among which there is TMEM9B. (**C**). Network of TMEM9B interactors, among which are ClC-3, -4, and -5, highlighted by green circles. (**D**). TMEM9B hydrophobicity plot showing a hydrophobic signal peptide (sequence positions 1–32) and a glycosylated asparagine at sequence position 60 in the extracellular/luminal domain. (**E**). TMEM9B AlphaFold predicted structure, highlighting, in cyan, the hydrophobic region from sequence positions 99 to 144, and, in magenta, the glycosylated asparagine at position 60. The signal peptide was removed from the AlphaFold structure and the image was created with PyMol.

Interrogating the Biogrid database of protein-protein interactions with physical evidence for interaction (https://thebiogrid.org (accessed on 7 August 2023)) with “CLCN3” and “CLCN4” as keywords revealed many putative interaction partners for ClC-3 (Figure 1A) and three for ClC-4 (Figure 1B). Interestingly, the database search revealed the membrane protein TMEM9B of unknown function as a possible physical interactor of ClC-3, -4, and -5 transporters (Figure 1C), but not the lysosomal ClC-7 antiporter. TMEM9B is a glycosylated type I transmembrane protein with a cleavable signal peptide (Figure 1D) with extracellular N-terminus and intracellular C-terminus [26]. The predicted AlphaFold structure [27] is shown in Figure 1E.

Little is known about TMEM9B. It appears to be localized in acidic intracellular organelles and has been implicated in the transcriptional control of cytokine production [26]. Several papers describe the role of enhanced TMEM9B expression in various types of cancer, possibly via interaction with the vesicular H^+^-ATPase (e.g., [29,30]). A *Tmem9b* knockout mouse showed no overt phenotype [29], but the analysis was focused on the involvement of the protein in cancer. According to the EMBL-EBI expression atlas (https://www.ebi.ac.uk/gxa/home (accessed on 1 March 2024)), the gene is expressed at a medium level in mouse and rat brains, suggesting a potential role in brain physiology.

### 3.2. Co-Expression of CLC Proteins with TMEM9B in Xenopus Oocytes

**Figure 2:**
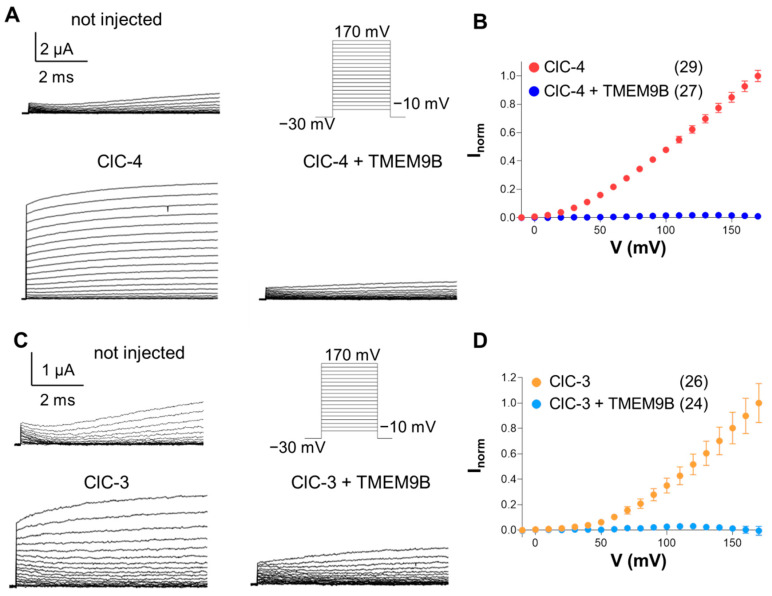
Co-expression of ClC-4 and ClC-3 with TMEM9B in *Xenopus* oocytes. (**A**). Representative recordings of non-injected oocytes and oocytes injected with ClC-4 and with ClC-4 + TMEM9B evoked by the voltage-clamp protocol are shown on the right. (**B**). Averaged normalized I-V relationships of ClC-4 with and without TMEM9B. Currents are normalized as described in Methods. (**C**). Typical voltage clamp current traces of non-injected oocytes and oocytes injected with ClC-3 and co-injected with ClC-3 + TMEM9B in response to the stimulation protocol shown on the right. (**D**). Averaged normalized I-V currents collected for ClC-3 compared with ClC-3 co-injected with TMEM9B. Note that average currents from non-injected oocytes from the same batches are subtracted in the I-V plots and that, for some data points, error bars are smaller than symbol size.

To investigate the interaction between ClC-4 and TMEM9B, we first employed *Xenopus* oocytes for two-electrode voltage-clamp recordings. Oocytes were injected with ClC-4 cRNA alone or co-injected with both ClC-4 and TMEM9B cRNA. ClC-4 expression resulted in large currents that increased in amplitude with increasing membrane voltage (Figure 2A). However, co-expression of TMEM9B completely inhibited these currents (Figure 2A). Figure 2B shows average I-V plots, demonstrating the strong inhibitory effect of TMEM9B.

Analogous results were obtained for ClC-3. Employing the same stimulation protocol, co-injection of TMEM9B with ClC-3 markedly reduced the currents at all voltages (Figure 2C,D).

**Figure 3:**
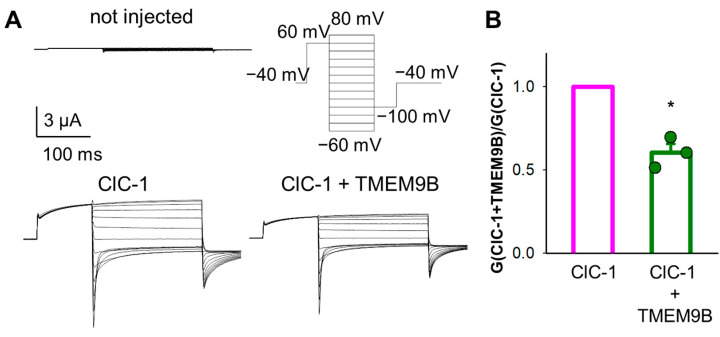
Co-expression of ClC-1 with TMEM9B in *Xenopus* oocytes. Typical current traces recorded from oocytes injected with ClC-1 alone and with TMEM9B (**A**). Stimulation protocol is shown as inset. (**B**) shows the normalized conductance of ClC-1 compared with ClC-1 with TMEM9B. For ClC-1, the slope conductance is the most robust parameter to quantify functional expression [20]. The error bar indicates SD (n = 3 injections). The star indicates *p* < 0.05 (Student’s *t*-test).

We next investigated the effect of TMEM9B on ClC-1-mediated currents (Figure 3A). Representative current traces are also shown. The normalized conductance of ClC-1 (Figure 3B) revealed a small but significant reduction upon co-expression with TMEM9B. While ClC-1 can be conveniently expressed in *Xenopus* oocytes, the large positive voltages and long pulse needed to activate ClC-7 frequently elicit endogenous currents. Therefore, we studied the effect of TMEM9B on ClC-7 in transfected cells (see below).

### 3.3. Co-Expression of CLC Proteins with TMEM9B in HEK Cells

We next tested the effect of TMEM9B on currents mediated by ClC-4, ClC-3, and ClC-7 in transfected HEK cells.

#### 3.3.1. Co-Expression of ClC-4 with TMEM9B in HEK Cells

**Figure 4:**
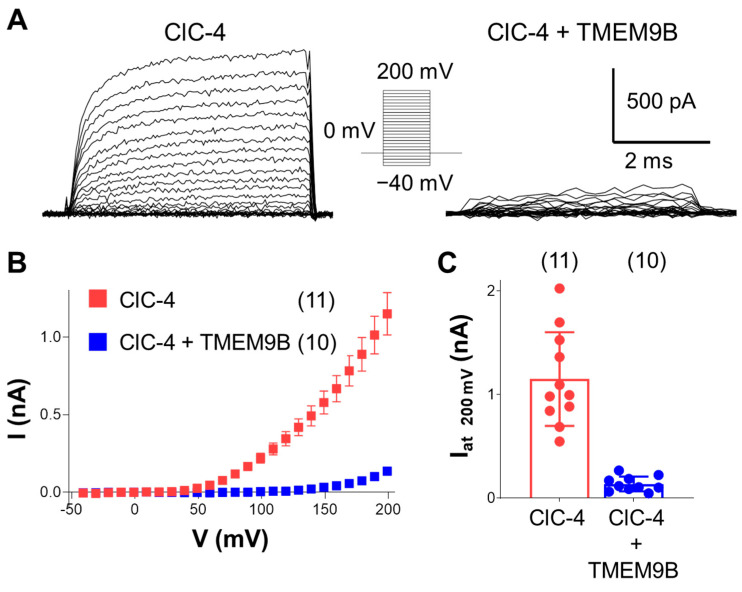
Co-expression with TMEM9B strongly reduced transport currents of ClC-4 in HEK cells. (**A**). Representative ionic currents elicited by the voltage-clamp protocol shown in the inset in control conditions (left trace) and in the presence of TMEM9B (right trace). (**B**). Average I-V plot shows a strong reduction of outward ClC-4 currents by TMEM9B (mean ± SEM). (**C**). Average current values at 200 mV (mean ± SD) (red bar, n = 11, I(200 mV) = 1.15 ± 0.45 nA; blue bar, n = 10, I(200 mV) = 0.14 ± 0.07 nA, *p* < 0.0001).

Whole-cell patch-clamp recordings show a strong reduction of outward ClC-4 mediated currents in cells expressing ClC-4, as well as TMEM9B, compared to cells expressing only ClC-4 (Figure 4), similar to the electrophysiological findings obtained in *Xenopus* oocytes.

#### 3.3.2. Co-Expression of ClC-3 with TMEM9B in HEK Cells

**Figure 5:**
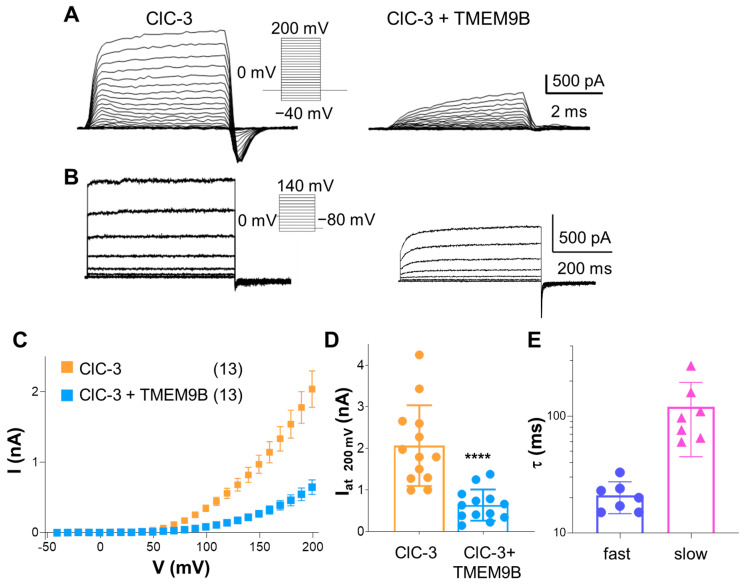
TMEM9B modulates the biophysical properties of ClC-3. (**A**). Representative ClC-3 currents elicited by the voltage-clamp protocol shown in the inset in control conditions (left trace) and in the presence of TMEM9B (right trace).0 (**B**). Representative recordings from a ClC-3 transfected cell (left trace) and from a cell co-transfected with TMEM9B using long (500 ms) pulses as indicated in the inset. (**C**). Average I-V plot in the absence (orange) and presence of TMEM9B (light blue, mean ± SEM). (**D**). Average current values at 200 mV (mean ± SD, orange bar, n = 13, I(200 mV) = 2.06 ± 0.97 nA; light blue bar, n = 13, I(200 mV) = 0.64 ± 0.37 nA; The four stars indicate *p* = 0.0002 (Student’s *t*-test). (**E**). Slowing of current activation by TMEM9B. Activation kinetics of cells co-transfected with TMEM9B was fitted with a double exponential function and values of the extracted time constants are shown as mean ± SD (purple bar: τ_fast_ =21.0 ± 6.4 ms; pink bar: τ_slow_ =120 ± 75 ms). No such slow kinetics were seen in cells transfected only with ClC-3.

Surprisingly, co-expression of ClC-3 with TMEM9B only partially reduced the activity of ClC-3 in HEK cells by about 70% (Figure 5A,C,D). Furthermore, interestingly, in the presence of TMEM9B, ClC-3 currents exhibited a slow activation time course, whereas ClC-3 alone activates very fast (Figure 5A). To get insights into the ClC-3 kinetic parameters affected by the interaction with TMEM9B, the pulse duration of the recording protocol was extended to 500 ms, followed by a “tail pulse” to −80 mV. The results confirmed that outward currents in the presence of TMEM9B exhibit a slow component in the activation kinetics (Figure 5B). Fitting the activation time course properly required a sum of two exponential components with time constants of 21.0 ± 6.4 ms and 120 ± 75 ms (Figure 5E). The biophysical alteration of ClC-3 kinetics strongly suggests a direct physical interaction between the proteins rather than an indirect effect on the number of transporters present in the membrane.

#### 3.3.3. TMEM9B Does Not Affect the Lysosomal ClC-7 Antiporter

**Figure 6:**
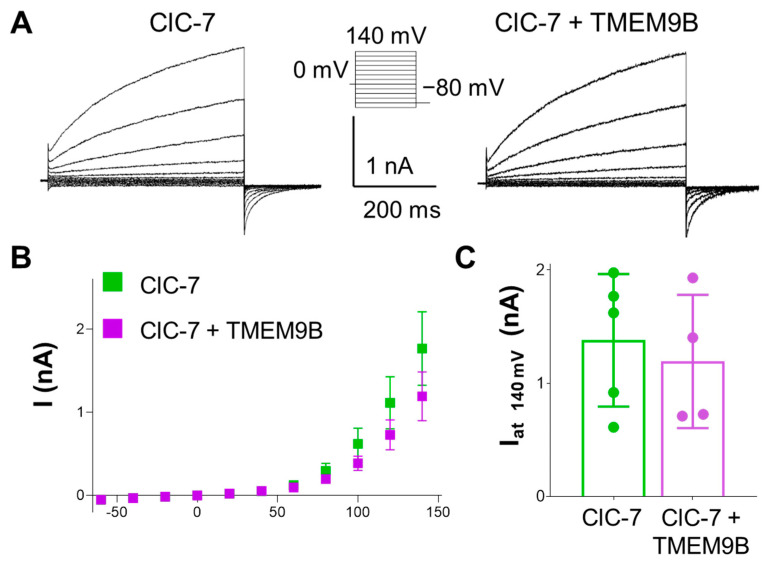
Co-expression with TMEM9B does not affect ClC-7 transport currents in HEK cells. (**A**). Representative ClC-7 currents elicited by the voltage-clamp protocol shown in the inset in control conditions (left trace) and in a cell co-transfected with TMEM9B (right trace). (**B**). Average I-V plot of ClC-7 transfected cells in the absence (green) and presence of TMEM9B (purple, mean ± SEM). (**C**). Average current values at 140 mV (mean ± SD, green bar, n = 5, I(200 mV) = 1.38 ± 0.58 nA; purple bar, n = 4, I(200 mV) = 1.19 ± 0.59 nA, *p* = 0.649).

To investigate the specificity of the interaction between TMEM9B and CLC members, functional characterization of the Ostm1/ClC-7 exchanger in the absence or in the presence of TMEM9B was performed. The outward currents carried by Ostm1/ClC-7 were very similar to those observed in cells co-expressing TMEM9B (Figure 6). This finding suggests that TMEM9B interacts specifically with the endosomal ClC-3 and ClC-4 proteins.

### 3.4. Subcellular Localization

**Figure 7:**
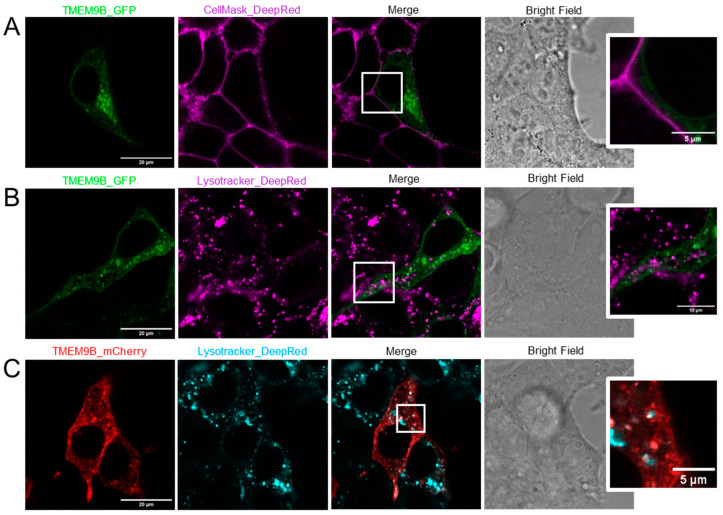
Subcellular localization of TMEM9B expressed alone. (**A**). Confocal images of HEK cells transfected with TMEM9B-GFP (green), stained with CellMask_DeepRed (magenta), merged image, and corresponding bright field image. The squared region is shown zoomed on the right. (**B**). Confocal images of cells transfected with TMEM9B-GFP (green) and stained with Lysotracker_DeepRed (magenta), merged image, and corresponding bright field image. The squared region is shown zoomed on the right. (**C**). Similar to B, but using mCherry tagged TMEM9B (red).

As already described by others, in HeLa cells, TMEM9B localized mainly in lysosomes, with partial endosome, perinuclear, and vesicular localization [26]. Using a different cell line (HEK), we first tested subcellular localization of TMEM9B alone (Figure 7). Representative confocal microscopy images of HEK cells transfected with TMEM9B-GFP (green) and stained with CellMask_DeepRed (magenta), together with the corresponding bright field image, are shown in Figure 7A. The merged confocal image reveals that TMEM9B is not localized in the plasma membrane, showing an average Pearson coefficient of co-localization of 0.22 ± 0.02 (SEM, n = 36). For example, the magnification of the squared area clearly shows that TMEM9B is localized intracellularly. Confocal images of cells transfected with TMEM9B-GFP (green) and stained with Lysotracker_DeepRed (magenta), together with the corresponding bright field image, are shown in Figure 7B. The merged image reveals a partial, but not exclusive, lysosomal localization, highlighted in more detail in the magnification of the squared field. The Pearson coefficient of co-localization of TMEM9B and Lysotracker was 0.34 ± 0.04 (SEM, n = 20). Similar results were obtained with mCherry tagged TMEM9B (red) stained with Lysotracker_DeepRed (cyan) (Figure 7C). Here, the Pearson coefficient of co-localization was 0.61 ± 0.07 (SEM, n = 10).

**Figure 8:**
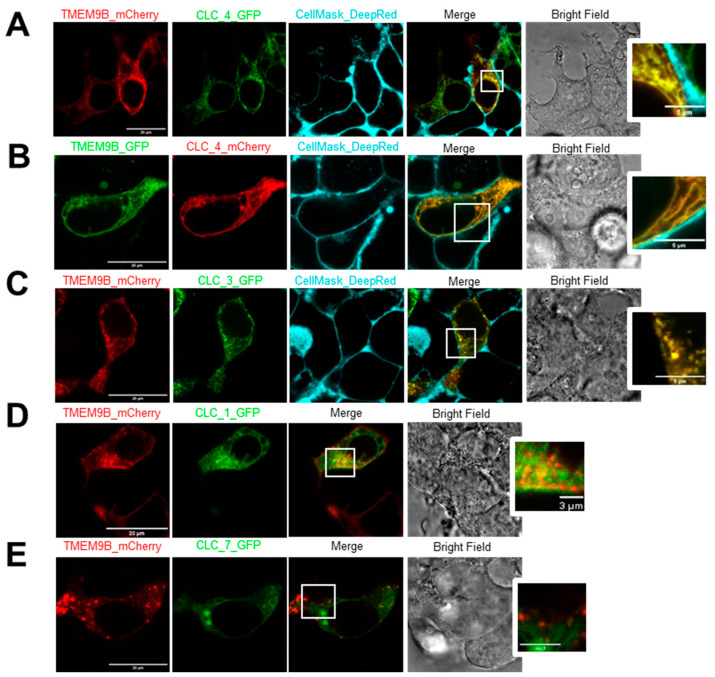
Subcellular localization of TMEM9B expressed with CLC proteins. (**A**). Confocal images of HEK cells co-transfected with TMEM9B-mCherry (red) and ClC-4-GFP (green), stained with CellMask_DeepRed (cyan), the merged image, and the corresponding bright field image. The squared region is shown zoomed on the right. (**B**). Similar results with inverted tags, i.e., TMEM9B-GFP (green) and ClC-4-mCherry (red). (**C**). Similar results for cells co-transfected with TMEM9B-mCherry (red) and ClC-3-GFP (green). (**D**). Similar results for cells co-transfected with TMEM9B-mCherry (red) and ClC-1-GFP (green). (**E**). Similar results for cells co-transfected with TMEM9B-mCherry (red) and ClC-7-GFP (green).

Furthermore, we tested subcellular localization of TMEM9B co-expressed with ClC-4, -3, and -7, respectively. In particular, representative confocal images of HEK cells co-transfected with TMEM9B-mCherry (red) and ClC-4-GFP (green), stained with CellMask_DeepRed (cyan) are shown in Figure 8A. The merged image reveals a prevalent reticular co-localization between TMEM9B and ClC-4. The Pearson coefficient of co-localization of TMEM9B and ClC-4 was 0.91 ± 0.01 (SEM, n = 99). The magnified view on the right clearly shows the absence of TMEM9B and ClC-4 on the plasma membrane. Figure 8B shows similar results with inverted fluorescent tags (Pearson coefficient: 0.92 ± 0.02 (SEM, n = 50)). The reticular localization is in good accordance with previous localization studies of ClC-4 [31]. We next studied co-localization with ClC-3. Representative images of cells co-transfected with TMEM9B-mCherry (red) and ClC-3-GFP (green), stained with CellMask_DeepRed (cyan), are shown in Figure 8C. The merged image shows intracellular vesicular, likely endosomal, and also plasma-membrane co-localization of TMEM9B with ClC-3. The magnified view of the squared field highlights such co-localization. The Pearson coefficient of co-localization was 0.82 ± 0.02 (SEM, n = 45). Thus, for both ClC-3 and ClC-4, it appears that localization of TMEM9B “follows” the localization of the respective co–expressed CLC transporter.

We also investigated co-localization of TMEM9B with ClC-1. Representative images of cells co-transfected with TMEM9B-mCherry (red) and ClC-1-GFP (green) are shown in Figure 8D. The merged image shows partial co-localization of TMEM9B with ClC-1, as shown in the zoomed view. The Pearson coefficient of co-localization was 0.62 ± 0.03 (SEM, n = 10).

As a negative control, we next investigated co-localization of TMEM9B with ClC-7. Representative images of cells co-transfected with TMEM9B-mCherry (red) and ClC-7-GFP (green) are shown in Figure 8E. The merged image shows only marginal co-localization of TMEM9B with ClC-7, highlighted in the zoomed view. The Pearson coefficient of co-localization was 0.36 ± 0.03 (SEM, n = 42).

### 3.5. FLIM-FRET Analysis of TMEM9B with CLC Proteins

**Figure 9:**
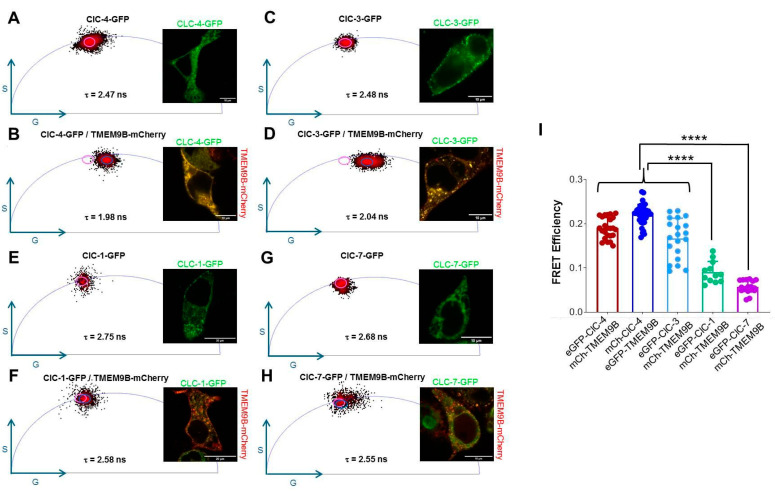
FLIM-FRET analysis of TMEM9B co-expressed with CLC proteins. (**A**). ClC-4-GFP phasor plot with corresponding lifetime value and representative image of a ClC-4-GFP transfected HEK cell. (**B**). ClC-4-GFP/TMEM9B-mCherry phasor plot with relative lifetime value and representative fluorescent confocal merged image of a ClC-4-GFP/TMEM9B-mCherry co-transfected HEK cell. Here, and in panels D, F, and H, the purple circle indicates the GS-coordinates of the unquenched donor. (**C**). ClC-3-GFP phasor plot with relative lifetime value and representative fluorescent confocal merged image of a ClC-3-GFP transfected HEK cell. (**D**). ClC-3-GFP/TMEM9B-mCherry phasor plot with relative lifetime value and representative fluorescent confocal merged image of a ClC-3-GFP/TMEM9B-mCherry co-transfected HEK cell. (**E**). ClC-1-GFP phasor plot with relative lifetime value and representative image of a ClC-1-GFP transfected HEK cell. (**F**). ClC-1-GFP/TMEM9B-mCherry phasor plot with relative lifetime value and representative image of a ClC-1-GFP/TMEM9B-mCherry co-transfected HEK cell. (**G**). ClC-7-GFP phasor plot with relative lifetime value and representative image of a ClC-7-GFP transfected HEK cell. (**H**). ClC-7-GFP/TMEM9B-mCherry phasor plot with relative lifetime value and representative image of a ClC-7-GFP/TMEM9B-mCherry co-transfected HEK cell. (**I**). FRET Efficiency analysis comparison with **** *p* < 0.0001 compared to the other groups.

While the localization studies described above suggest co-localization of TMEM9B with ClC-3 and ClC-4, the resolution of confocal microscopy is not sufficient to prove direct physical interaction. Förster Resonance Energy Transfer (FRET) is an energy transfer process between a fluorescent donor and a nearby acceptor fluorophore which is highly sensitive to the distance between donor and acceptor. To test for direct physical interaction between TMEM9B and ClC-3 and ClC-4, we transfected HEK cells with ClC-3/-4/-1/-7 and TMEM9B, each fused to the green fluorescent protein, GFP, or to the red fluorescent protein, mCherry, in different combinations and measured the fluorescent lifetime as described in Methods. The phasor plots with corresponding lifetime values and representative fluorescent confocal merged image of a cell for each condition are shown in Figure 9. The lifetime value of ClC-4-GFP alone was significantly reduced by co-expression with TMEM9B (Figure 9A,B), with the resulting co-localized as shown in the fluorescent confocal merged image (yellow) and in a relatively large average FRET efficiency of about 0.19 (Figure 9I). Also, the inverse FRET pair TMEM9B-GFP/ClC-4-mCherry showed a high FRET efficiency value (Figure 9I). Similarly, the lifetime value of ClC-3-GFP was reduced upon co-expression with TMEM9B-mCherry (Figure 9C,D), with the resulting co-localized as shown in the fluorescent confocal merged image (yellow) and in an averaged FRET efficiency of about 0.17 (Figure 9I). These data strongly suggest direct physical interaction between ClC-3/-4 and TMEM9B. In contrast, neither for ClC-1 (Figure 9E,F) nor for ClC-7 (Figure 9G,H) co-expression with TMEM9B-mCherry reduced the donor-lifetime value as much. For ClC-1, an average FRET efficiency of 0.09 was calculated (Figure 9I), slightly larger than for ClC-7, for which a value of 0.06 was obtained (Figure 9I).

## 4. Discussion

Many ion channel and transporter proteins are associated with regulatory or stabilizing accessory subunits and CLC proteins are no exception: CLC-K channels associate with barttin in an obligatory manner [14], ClC-7 requires Ostm1 [15], and ClC-2 associates with GlialCAM in glial cells [16]. So far, no accessory protein was known for the endosomal CLC sub-branch that comprises the highly homologous members ClC-3, -4, and -5. Several proteins are listed in interaction databases as possible partners of ClC-3 and ClC-4, but the relevance of such database entries requires further validation. Here, we discovered that one such candidate, TMEM9B, a protein of unknown function, tightly interacts with and modifies the function of the neuronal endosomal Cl^−^/H^+^ antiporters ClC-3 and ClC-4. The physiological role of TMEM9B is unknown, but several papers describe a role of enhanced TMEM9B expression in several types of cancer, possibly via interaction with the vesicular H^+^-ATPase (e.g., [29,30]). A *Tmem9b* knockout mouse showed no overt phenotype [29], but the analysis concentrated on the involvement of the protein in cancer.

We provide a multitude of data that clearly show that TMEM9B specifically interacts with ClC-3 and ClC-4 by strongly reducing their function. Firstly, co-expression in *Xenopus* oocytes completely inhibited ClC-3, as well as ClC-4 mediated currents. Second, even if ClC-3 was less inhibited in transfected HEK cells, TMEM9B induced a slow component in the activation kinetics of ClC-3, strongly suggesting direct physical interaction. Third, ClC-7 mediated currents were hardly affected and ClC-1 currents were much less reduced. The possible physiological significance of the interaction of TMEM9B with ClC-1 remains to be investigated.

The reduction of ClC-3 and ClC-4 mediated currents upon co-expression with TMEM9B might be mediated by a reduction of plasma membrane expression, for example, by retention or by increased endocytosis. However, the functional alteration of ClC-3 seen in HEK cells and the presence of ClC-3-GFP at the level of the plasma membrane in TMEM9B co-transfected cells suggests that this is unlikely to be the only mechanism underlying the current reduction.

Further evidence for interaction between ClC-3/-4 and TMEM9B was obtained by confocal co-localization. TMEM9B alone localized mostly intracellularly, but not exclusively in lysosomes, in contrast to a previous report [26]. Co-expression with ClC-3 and ClC-4 showed extensive overlap, and it appeared that the localization of TMEM9B was determined by that of ClC-3/-4, similar to what has been shown for Ostm1 and ClC-7 [15].

Moreover, using Förster Resonance Energy Transfer (FRET), we showed direct physical interaction between fluorescent tags on ClC-3/-4 and TMEM9B, respectively. For the couple GFP-mCherry, the Förster radius, R_0_, is around 5 nm [32], such that FRET occurs only if the two proteins are at a distance within this range. We used fluorescence lifetime imaging microscopy to detect FRET via shortening of the donor lifetime. In agreement with the functional data, we detected significant FRET between TMEM9B and ClC-3, and ClC-4, but hardly any FRET with ClC-1 or ClC-7, showing again that the interaction is specific.

An interesting result is that the FRET efficiency of the pair ClC-4-GFP/TMEM9B-mCherry was similar to that of the pair with inverted fluorophores, i.e., TMEM9B-GFP/ClC-4-mCherry. This result sheds light on the possible stoichiometry of the TMEM9B-CLC complex. In fact, if, for example, more than one TMEM9B protein would associate with a single CLC monomer, the FRET efficiency in the TMEM9B-GFP/ClC-4-mCherry complex would be expected to be much smaller than in the inverted ClC-4-GFP/TMEM9B-mCherry complex, because each donor would have fewer acceptors to which energy can be transferred. The same argument holds for the inverse case. Thus, the mere fact that the two inverse couples of tags exhibited similar FRET efficiency strongly suggests that the two proteins are associated in a 1:1 stoichiometry. Since CLC proteins are dimers, this might imply that two TMEM9B subunits are present in each CLC dimer. Since the membrane topology of TMEM9B is similar to that of Ostm1, i.e., both have an N-terminal luminal domain and a cytoplasmic C-terminus, it can be speculated that TMEM9B associates with ClC-3/-4 in a similar manner as does Ostm1 with ClC-7 [33,34].

The discovery of the novel interactor TMEM9B of the endosomal Cl^−^ transporting ClC-3 and ClC-4 could be crucial for a better understanding of the physiology of these proteins and the pathologies caused by mutations in the genes encoding these transporters [7,12,13]. For example, it will be interesting to study the possible effects of disease-causing variants on the interaction between ClC-3/-4 and TMEM9B. It will also be interesting to explore the possible physiological relevance of the alteration of biophysical properties of ClC-3 mediated currents by TMEM9B, and whether such alteration occurs in ClC-3/ClC-4 heterodimers.

While our data of a specific interaction between TMEM9B and ClC-3/-4 strongly suggest that the interaction we found in vitro is physiologically relevant in vivo, this still needs to be demonstrated. A crucial further question is whether and how the interaction of TMEM9B with ClC-3/-4 is regulated. The fact that we observe, in vitro, a strong down-regulation of ClC-3 and ClC-4 mediated activity upon co-expression with TMEM9B suggests that the accessory protein could act as a negative regulator in vivo. Such an activity is unlikely to be constitutive but is probably regulated by specific cellular needs. The investigation of such regulatory mechanisms will be a future challenge.

Moreover, it will be interesting to test whether TMEM9B interacts with the kidney-specific ClC-5 transporter, which is involved in Dent’s disease [35,36]. ClC-5 shares high sequence identity with ClC-3 and ClC-4 and has very similar functional properties [37]. Also, from the NCBI database (https://www.ncbi.nlm.nih.gov/gene/56674 or https://www.ncbi.nlm.nih.gov/gene/56786 (accessed on 5 June 2024)), TMEM9B is expressed in human and mouse kidney at a medium level. Similarly, further studies are needed to investigate in more detail a possible interaction of TMEM9B with the skeletal muscle specific ClC-1 channel. While we found a significant reduction of currents, and also considerable co-localization, only a rather small FRET signal could be detected.

Finally, TMEM9 is another protein listed in interaction databases as a candidate partner of ClC-3 and ClC-4 (see also Figure 1). TMEM9 and TMEM9B share 57% sequence identity, and also TMEM9 has a cleavable signal peptide [38]. We speculate that TMEM9 interacts with CLC proteins in a similar manner as TMEM9B.

In conclusion, our results open new directions in the investigation of the pathophysiological role of ClC-3 and ClC-4 with their interacting accessory proteins.

## Data Availability

Primary data shown in the Figures will be made available upon reasonable request.
